# TSPYL2 Regulates the Expression of EZH2 Target Genes in Neurons

**DOI:** 10.1007/s12035-018-1238-y

**Published:** 2018-07-26

**Authors:** Hang Liu, Lei Peng, Joan So, Ka Hing Tsang, Chi Ho Chong, Priscilla Hoi Shan Mak, Kui Ming Chan, Siu Yuen Chan

**Affiliations:** 1 Department of Paediatrics and Adolescent Medicine, Li Ka Shing Faculty of Medicine, The University of Hong Kong, Hong Kong, China; 2 Present Address: University Research Facility in Chemical and Environmental Analysis, The Hong Kong Polytechnic University, Hong Kong, China; 3 Present Address: Peter MacCallum Cancer Centre, Melbourne, VIC Australia; 4 Present Address: Research and Development, Clinical Projects and Development, New B Innovation, Hong Kong, China; 5 Department of Biomedical Sciences, the City University of Hong Kong, Hong Kong, China; 6 Key Laboratory of Biochip Technology, Biotech and Health Centre, Shenzhen Research Institute of City University of Hong Kong, Shenzhen, China

**Keywords:** Nucleosome assembly protein, Epigenetics, TSPYL2, H3K27me3, EZH2, Neurons

## Abstract

*Testis-specific protein*, *Y-encoded-like 2* (TSPYL2) is an X-linked gene in the locus for several neurodevelopmental disorders. We have previously shown that *Tspyl2* knockout mice had impaired learning and sensorimotor gating, and TSPYL2 facilitates the expression of *Grin2a* and *Grin2b* through interaction with CREB-binding protein. To identify other genes regulated by TSPYL2, here, we showed that *Tspyl2* knockout mice had an increased level of H3K27 trimethylation (H3K27me3) in the hippocampus, and TSPYL2 interacted with the H3K27 methyltransferase enhancer of zeste 2 (EZH2). We performed chromatin immunoprecipitation (ChIP)-sequencing in primary hippocampal neurons and divided all Refseq genes by k-mean clustering into four clusters from highest level of H3K27me3 to unmarked. We confirmed that mutant neurons had an increased level of H3K27me3 in cluster 1 genes, which consist of known EZH2 target genes important in development. We detected significantly reduced expression of genes including *Gbx2* and *Prss16* from cluster 1 and *Acvrl1*, *Bdnf*, *Egr3*, *Grin2c*, and *Igf1* from cluster 2 in the mutant. In support of a dynamic role of EZH2 in repressing marked synaptic genes, the specific EZH2 inhibitor GSK126 significantly upregulated, while the demethylase inhibitor GSKJ4 downregulated the expression of *Egr3* and *Grin2c*. GSK126 also upregulated the expression of *Bdnf* in mutant primary neurons. Finally, ChIP showed that hemagglutinin-tagged TSPYL2 co-existed with EZH2 in target promoters in neuroblastoma cells. Taken together, our data suggest that TSPYL2 is recruited to promoters of specific EZH2 target genes in neurons, and enhances their expression for proper neuronal maturation and function.

**Electronic supplementary material:**

The online version of this article (10.1007/s12035-018-1238-y) contains supplementary material, which is available to authorized users.

## Introduction

*Testis-specific protein*, *Y-encoded-like 2* (TSPYL2) belongs to the nucleosome assembly protein (NAP) superfamily, which is a poorly studied group of histone chaperons [1–3]. The NAP superfamily is divided into three families of NAP1-like (NAP1L), SET oncoprotein, and TSPYL [4]. So far, gene knockout has been reported for NAP1L2 and TSPYL2 in mice [5–8]. Targeted mutation of *Nap1l2* results in abnormal proliferation of neural progenitor cells and the phenotype resembles spina bifida [5]. By contrast, targeted mutation of *Tspyl2* results in impaired hippocampal long term potentiation and behavioral phenotypes [6, 9, 10]. Our group has shown that TSPYL2 directly regulates the transcription of genes for NMDA receptor subunits 2A (*Grin2a*) and 2B (*Grin2b*) in mice [9]. These functional studies provide evidence for the contribution of *TSPYL2* in neurodevelopmental disorders linked to its chromosome location Xp11 in humans [11–14]. As TSPYL2 is expressed in both neuronal progenitors and mature neurons [15], it may regulate the expression of multiple genes important for proper development and function of neurons.

One way NAPs regulate gene expression is by bridging the transcriptional regulatory complex and chromatin through histone binding [16]. In vitro evidences suggest that NAP1L1, NAP1L4, and SET interact with histone acetyltransferases p300 and CREB-binding protein (CBP) [16–18]. Furthermore, NAP1 can work with CBP to facilitate transcription by promoting nucleosome eviction at the promoter [18]. Our group has detected interaction between TSPYL2 and CBP by a mammalian-two-hybrid assay [9]. From a proteomic study designed to detect weak or transient protein interactions of transcriptional co-regulators, TSPYL2 interacts with p300 and the transcriptional co-regulator complex “Z3,” which consists of *Z*MYND8, *Z*NF687, and *Z*NF592 [19]. ZMYND8 contains domains as a reader of methylated lysine residues of histone [20]. In carcinoma cells, TSPYL2 is an essential component of the repressor element 1 silencing transcription factor (REST) transcription complex. The study also identified various interacting partner of TSPYL2, including SIN3 and subunits of the nuclear remodeling NuRD complex (LSD1, MTA1, MTA2, RbAp46, and RbAp48) [21]. Therefore, we hypothesize that TSPYL2 interacts transiently with various transcription regulatory complexes and regulates gene expression in neurons.

Post-translation modifications of histone tails play a pivotal role in regulating gene expression. In general, histone acetylation at lysine positions, such as H3K9, 14, and 56, signifies transcription initiation [22, 23]; trimethylation at H3K36 is a hallmark of transcription elongation [23], while H3K27 trimethylation (H3K27me3) is a key repressive histone mark which plays an important role during brain development, neuron specification, and function [24–27]. The H3K27 methylation mark is set up by the polycomb repressive complex 2 (PRC2) [28, 29]. Mammalian PRC2 consists of four core components: enhancer of zeste (EZH), embryonic ectoderm development (EED), suppressor of zeste 12 homolog (SUZ12), and RbAp46/48 [30]. The presence of other components is context-dependent. For example, JARID2 forms part of the PRC2 complex and facilitates its recruitment to target genes in embryonic stem cells [31, 32], whereas in teratocarcinoma cells, REST can recruit PRC1 and PRC2 to target genes [33].

The main function of PRC2 is the maintenance rather than establishment of transcription silencing through chromatin compaction [34]. Although the level of EZH2 declines in post-mitotic neurons, recent evidences suggest a new role of EZH2 in regulating the expression of synaptic genes. The level of H3K27me3 at the *Grin2b* promoter increases during the first 2 months of post-natal development in rats, which is important for the developmental switch in the expression of *Grin2b* to *Grin2a* [35]. EZH2 also binds to the promoter of the gene for brain-derived neurotrophic factor (BDNF), but is released upon activation of primary neurons [36, 37]. Furthermore, the change in H3K27 methylation is associated with changes in the expression of specific transcript variants of *BDNF* in both humans and mice [38, 39].

Based on the expression of TSPYL2 in the developing and adult brain [7, 15] and its interaction with diverse histone modifiers and readers, we examined the effect of *Tspyl2* loss-of-function on histone modifications, which are correlated with gene activity. Intriguingly, we discovered an increased level of H3K27me3 in the mutant hippocampus. Since we found that TSPYL2 pulled down EZH2, we mapped H3K27me3 and EZH2 marked genes in primary hippocampal neurons using chromatin immunoprecipitation-sequencing (ChIP-seq). We further identified marked genes with significantly reduced expression in mutant neurons. Our data show that TSPYL2 is important for fine tuning the expression of important neuronal genes including *Bdnf*, *Egr3*, and *Grin2c2* and provide novel molecular insights into the behavioral changes in *Tspyl2* mutant mice.

## Materials and Methods

### Mice

Mouse experiments were performed in accordance with the relevant guidelines and regulation of the Laboratory Animal Unit at the University of Hong Kong, a facility accredited by the Association for Assessment and Accreditation of Laboratory Animal Care. Experiments were approved by the Committee on the Use of Live Animals in Teaching and Research at the University of Hong Kong (approval no. 3273-14). Detailed procedures for the generation and genotyping of *Tspyl2* loss-of-function mutant mice on a pure 129/SvEv genetic background have been described [7].

### Cell Culture

HEK293 cells were cultured in DMEM supplemented with 10% FBS and Be(2)-C cells in DMEM/F12 supplemented with 10% FBS. Cells were transfected with Fugene HD (Promega Corporation). Primary hippocampal neurons were isolated from 16.5-days mouse embryos and seeded in neurobasal medium (Invitrogen) supplemented with 1% FBS, B27 (Invitrogen), and penicillin/streptomycin on poly-D-lysine-coated culture dishes at days in vitro (DIV) 0. Glutamate (25 μM) was added at plating only, and half of the medium was replaced by medium without glutamate the next day and every 4 days afterwards. For drug treatment, 0.5-μM GSK126 (Selleckchem) was dissolved in DMSO and applied from DIV 7, replaced with fresh drug containing medium at DIV 10, and samples were collected at DIV 14. For GSK J4 treatment, GSK J4 (Selleckchem) in DMSO was applied at 0.1 μM at DIV 7 and samples were collected at DIV 10 before neurons showed morphological signs of suboptimal growth.

### Plasmids

Full length UTX and JMJD3 cDNAs were PCR amplified from a human liver cDNA library. The FLAG tag was introduced through the downstream PCR primer, and the cDNAs were subcloned into pBabe which allowed expression in HEK293 cells. Cloning of hemagglutinin (HA)-tagged TSPYL2 in pcDNA3 has been described [9]. The full-length TSPYL2 cDNA was inserted into pGEX-4T1 (GE Healthcare Life Sciences) to generate pGEX-GST-TSPYL2 for production of bacterial recombinant proteins. All plasmids were verified by sequencing.

### Western Blotting

Brain samples were removed from 2-month-old male littermate mice in ice-cold HBSS (Sigma). Hippocampi were collected and lysed with a Dounce homogenizer in IP buffer (10-mM Tris, pH 8.0, 1-mM EDTA, 1-mM EGTA, 150-mM NaCl, 0.5% Nonidet P-40, 1% Triton X-100) supplemented with complete protease inhibitors (Roche) and 100-μM MG132 (Sigma). Proteins (50 μg) were resolved and detected with standard immunoblotting procedures and ECL reagents (Millipore). Quantitation of the protein bands was done with software from GeneTools (Syngene). For cultured cells, proteins were collected in IP buffer with protease inhibitors and freeze-thaw once. The following primary antibodies were used: rabbit anti-H3K9, 14Ac (Cell Signaling, #9677), rabbit anti-H3K56Ac (Cell Signaling, #4243), rabbit anti-H3K27me3 (Cell Signaling, #9755), rabbit anti-H3K36me3 (Cell Signaling, #9758), rabbit anti-H3 (Cell Signaling, #4499), rabbit mAb anti-EZH2(D2C9) (Cell Signaling, #5246), mouse anti-Flag (M2) (Sigma, P3165), mouse anti-β tubulin (Upstate, 05-661), and goat anti-lamin B (Santa Cruz, sc-6220).

### Pull Down Assay

The production of GST in *BL21* was as described by GE Healthcare. Production of GST-TSPYL2 was optimized as follows. After IPTG induction, the total cell lysate was prepared by incubation in lysis buffer (PBS pH 7.5, 2-mM DTT, 1-mM PMSF) and freeze-thaw three times. Triton X-100 was added to the final concentration of 1%, and the lysate was purified with Glutathione Sepharose 4B following the manufacturer’s instructions (GE Healthcare), with the addition of 2-mM DTT and 0.5% Triton X-100 in the washing buffer. For pull down assays, nuclear lysates from 5 × 10^6^ HEK293 cells were resuspended in Buffer A (10-mM HEPES pH 8.0, 1.5-mM MgCl_2_, 10-mM KCl, 0.5-mM DTT, 0.05% NP40). After centrifugation, the pellet (nuclear fraction) was lysed in buffer B (5-mM HEPES pH 8.0, 1.5-mM MgCl_2_, 0.2-mM EDTA, 0.5-mM DTT, 26% glycerol *v*/*v*, 312.5-mM NaCl), incubated 30 min on ice with vortex at 10-min intervals, and centrifuged to remove debris. Nuclear lysates (150 μg) were added onto the washed Glutathione Sepharose 4B beads with GST or GST-TSPYL2 and incubated for 3 h at 4 °C with rotation. The beads were washed four times with IP buffer and boiled in sample buffer for western blot analysis.

### Far-Western Assay

To elute the recombinant proteins, washed Glutathione Sepharose 4B beads with GST or GST-TSPYL2 produced as above were incubated at room temperature for 10 min with 10-mM glutathione in 50-mM Tris-HCl pH 8.0. EZH2 complex consisting of EZH2, EED, SUZ12, RbAp48, and AEBP2 (BPS Bioscience) was loaded in triplicate of 0.2 μg per well of a 7.5% polyacrylamide gel, separated, and transferred to a nitrocellulose membrane. The triplicate lanes were cut separate and blocked with 3% BSA. One strip was probed with rabbit mAb anti-EZH2 (D2C9) for western blot. The other two strips were incubated in either 0.6-μg GST or 2-μg GST-TSPYL2 in 5-ml TBST with 3% BSA overnight at 4 °C with rotation. After incubation, the membranes were probed by GST antibody (GE Healthcare, #27-4577-01) following standard procedures for western blot.

### Immunocytochemistry

Cells were plated onto coverslip coated with 0.1% gelatin in a 24-well. The coverslips were picked up and cells were fixed in 4% PFA for 10 min, washed by PBS for three times, and blocked by 5% normal goat serum in PBST for 1 h. Cells were incubated with primary antibodies in 5% normal goat serum in PBST and washing three times in PBST. The primary antibodies were mouse anti-NeuN (Chemicon, MAB377) and rabbit anti-Ezh2 (Cell Signaling, D2C9). Signals were visualized with Alexa Flour 488 or 594 labeled-goat antibodies (Life Technologies) and counterstaining with DAPI (Sigma). Coverslips were mounted onto slides and examined under the epifluorescence microscope (Carl Zeiss, Axioplan 2 imaging).

### Hybridization to Peptide Arrays

For testing the specificities of H3K27me3 antibodies for ChIP (Diagenode, pAb-069-050), antibodies were applied to EpiTitan Histone Peptide Array (Epicypher, #11-2001), washed three times with PBST (PBS pH 7.4, 0.1% Triton-X 100), then hybridized with Alexa Fluor-594 goat anti-rabbit secondary antibodies (Life Technologies), and signals were detected by a Typhoon Scanner (GE Healthcare Life Sciences).

### ChIP-seq

Wild-type and mutant primary neurons were collected and cultured in parallel. One thousand cells were seeded per mm^2^ at DIV 0. Neurons were cross-linked by 1% formaldehyde for 10 min and harvested in lysis buffer with protease inhibitors. DNA was fragmented using Biorupter (Diagenode) with 18 cycles of 30 s on and 60 s off. The DNA fragments were mainly at 300–500 bp as checked by fluorometric quantitation (Qubit). An aliquot of chromatin from one million cells were used for each IP, washed once with IP buffer, twice with low salt wash buffer (0.1% SDS, 1% Triton X-100, 2-mM EDTA, 20-mM Tris-HCl pH 8.0, 150-mM NaCl), once with high salt buffer (0.1% SDS, 1% Triton X-100, 2-mM EDTA, 20-mM Tris-HCl pH 8.0, 500-mM NaCl), and once with TE buffer containing 50-mM NaCl. All antibodies used were ChIP grade: H3K27me3 (Diagenode, pAb-069-050) and EZH2 (D2C9) rabbit mAb (Cell Signaling, #5246). DNA was eluted and purified according to X-ChIP protocol (Abcam). After ChIP, purified DNA samples were size-selected (around 300 bp), and libraries were constructed with NEBNext® ChIP-Seq Library Prep Reagent Set (NEB) and sequenced by the Genome Analyzer IIx System and HiSeq1500 (Illumina). Sequencing data was mapped to mouse mm9 genome using BWA with default parameters [40]. MASC 2.0 was used for generating tags pileup [41]. Pileup files were scaled to one million mappable reads for downstream analysis. Gene annotations (refGene.txt.gz) were downloaded from UCSC repository. The longest transcript of each gene was selected for analysis. Metagene analysis was performed using customized Perl and R scripts. The H3K27me3 modification level was averaged in every 100-bp bins from 5-kb up- and down-stream of TSS for k-mean clustering.

### RT-qPCR

RNA was extracted using Trizol or RNAzol (Invitrogen). Two μg of total RNA was used for reverse transcription in 20 μl using oligo(dT) and reverse transcriptase (Invitrogen). Quantitative PCR was done with 0.5 μl of cDNA by using QuantiFast SYBR green PCR kit (Qiagen) in 7900HT Fast Real-Time PCR System (Applied Biosystems). The expression level of *Hprt* is within two-cycle difference to that of neuronal genes, for example *Bdnf* and *Grin2b* in RT-qPCR, and therefore, we used it as the internal control. Relative expression was determined by the ΔΔCt method. Sequences of primers (5′ → 3′) were:

Hprt-F(AACTGGAAAGAATGTCTTGATTG) and Hprt-R(TCAAATCCAACAAAGTCTGGC); Acvrl1-F (CAGACACCCACCATCCCTAA) and Acvrl1-R(TGGGGTACCAGCACTCTCTC); Bdnf-E1F(TGCATCTGTTGGGGAGACAAG) and Bdnf-E9R (CACACCTGGGTAGGCCAAG); Cldn2-F(TGTTGGTGCCAGCATTGTGA) and Cldn2-R(TGTCCTTAGCTCGAGAATCC); Egr2-F(GGCCGTAGACAAAATCCCAG) and Egr2-R(AGGGTACTGTGGGTCAATGG); Egr3-F (GACCAACGAGAAGCCCAATC) and Egr3-R(GTTGGAATAAGGAGGCAGCG); Gbx2-F (CAACTTCGACAAAGCCGAGG) and Gbx2-R (CTTGCCCTTCGGGTCATCTT); Grin2a-F(CATCAGCAGGGGCATCTACA) and Grin2a-R (CCCTTGGACTCATTGAGAGT); Grin2b-F (CCATCAGCAGAGGTATCTAC) and Grin2b-R (CAGTCTGAATGCGTGAAGCT); Grin2c-F(TGGCTCAAACACAGTCGTTG) and Grin2c-R (CTGTAGCCTGTACCTAGCCC); Grik3-F(TACAGGTCTCATCCGTCTGC) and Grik3-R(CCCGCTTCATCTCTTTGAGC); Igf1-F (ACTGGAGATGTACTGTGCCC) and Igf1-R (GATAGGGACGGGGACTTCTG); Klf4-F (GAAATTCGCCCGCTCCGATGA) and Klf4-R (CTGTGTGTTTGCGGTAGTGCC); Med12-F(GTGGCTATGTGCATCAGCAG) and Med12-R(GCACTCAACGGAGTCATGGT); Otx2-F(CGTTCTGGAAGCTCTGTTTG) and Otx2-R (GCCACTTGTTCCACTCTCTGA); Pax6-F (ACCCAAGAGCAGATTGAGGC) and Pax6-R(GCCTGTCTTCTCTGGTTCCTC); Prss16-F (GAACCGCAAGTGTCTGGTGT) and Prss16-R (GCTGGGAAAAAGGACACTGA); Skor2-F (CACAGACAAGAGCCCTCAGG) and Skor2-R (TTGACGGAACTGAGTGGGTG); Slc27a2-F(AACACATCGCGGAGTACCTG) and Slc27a2-R (TTGAAGCCCTCTTCCATCAG).

### ChIP-PCR

Cells were transfected with Fugene HD (Promega Corporation). After 48 h, ChIP was performed according to the X-ChIP protocol (Abcam) with minor modifications. Briefly, after cell harvesting, 250-μl cell lysates were sonicated as for primary neurons and checked by electrophoresis to give mainly small fragments of 300–500 bp. Lysates were immuno-precipitated with 2-μg mouse anti-HA antibodies (Sigma, H3663) overnight, followed by adding 20-μl DiaMag protein A-coated magnetic beads for 2 h. The complex was washed three times with IP buffer, once with low salt wash buffer and once with TE plus 50-mM NaCl. DNA was eluted and purified according to X-ChIP protocol. Input (3.5%) was used for comparison. DNA fragments were detected by PCR using Hotstart Taq (Qiagen) with specific primers. The sequences of ChIP Primers (5′ → 3′) were:

GBX2-F (TGCTAACTGCAGGATGGAGC) and GBX2-R (CTTGGAGACTCGAGGAAGCC); PRSS16-F (ATGAATCTGTTGGGCTGCGA) and PRSS16-R (TGGGTTTGCTTCCAGTACCC); BDNF-F (GCATTCAGTGGCTGCTTCAAGG) and BDNF-R (GGGCTGCAATGAACAGAGGTC); GRIN2A-F (GCTTAGAAGTGGCAGCCTCA) and GRIN2A-R(AATGCTTCTCCCTGTGCCTC); ACTB-F (GCGCCGCTGGGTTTTATAGG) and ACTB-R (CTCCTCTTCCTCAATCTCGCTC).

### Statistical Analysis

Gene Ontology enrichment analysis was done at DAVID Gene Ontology Bioinformatic Resources (https://david.ncifcrf.gov/home.jsp), and cutoff was set to 0.05 for *P* value (a modified Fisher’s exact *P* value) adjusted by Benjamini-Hochberg correction.

For RT-qPCR analysis of untreated primary hippocampal neurons, each set of samples consisted of a litter of five to six embryos (i.e., *n* = 5 or 6) collected in parallel from one wild-type and one mutant females. A total of two sets of embryos were collected. The mean ΔCt of wild-type, here designated as ΔCt_mean_, was used for normalization. The relative expression in WT was shown as 2^−(∆Ct^_WT_^−∆Ct^_mean_^)^. The relative expression in mutant was calculated according to 2^−(∆Ct^_KO_^−∆Ct^_mean_^)^. Data were analyzed using Student’s *t* test by Prism software. For drug treatment, hippocampal neurons collected from each embryo were divided equally onto two wells of a 12-well plate, with one well receiving vehicle (*v*) and one well receiving the drug (*d*). The relative expression in the vehicle side was set as 1. The relative expression in drug treated side was calculated according to 2^−(ΔCt^_d_^−ΔCt^_v_^)^. Data were analyzed using paired *t* test by Prism.

## Results

### TSPYL2 Affects the Level of H3K27me3 in Hippocampal Neurons

**Fig. 1:**
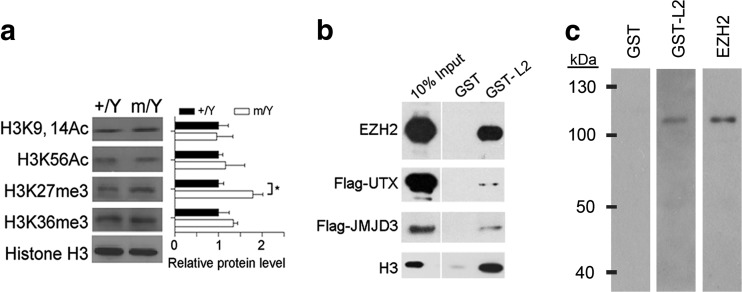
TSPYL2 reduces trimethylation of H3K27 and interacts with EZH2. **a** Left: representative western blot of hippocampal lysates collected from 2-month-old male littermates. Right: a specific upregulation of H3K27me3 in the mutant hippocampus. Relative protein level to H3 in the wild-type was set as 1. *n* = 4 per genotype. Error bars represent SEM. *P < 0.05, Student’s *t* test. **b** GST-tagged TSPYL2 (GST-L2) pull-down using nuclear lysates from non-transfected (first and bottom rows), Flag-UTX-transfected (second row), and Flag-JMJD3-transfected HEK293 cells (third row). Western blot for proteins indicated on the left. **c** Components of PRC2 complex (EZH2, EED, SUZ12, RbAp48, and AEBP) were separated by a polyacrylamide gel, probed with recombinant GST, GST-L2, or antibodies to EZH2 as indicated on the top

We tested whether the *Tspyl2* loss-of-function would affect the levels of well-known chromatin marks associated with transcription as described in introduction. Intriguingly, we detected a specific upregulation of H3K27me3, a key histone modification important for brain development and neuron function, in the mutant hippocampus by western blot (Fig. 1a). To test whether TSPYL2 directly affects the methylation of H3K27, we performed affinity pull down using bacterial recombinant GST-tagged TSPYL2 with HEK293 nuclear extract. We detected EZH2 and interestingly, only weak signals of the H3K27 demethylases UTX and JMJD3 were detected even when they were overexpressed individually (Fig. 1b). To test if the interaction between TSPYL2 and EZH2 was direct or indirect, we separated components of the PRC2 core complex consisting of EZH2, EED, SUZ12, RbAp48, and AEBP and probed with recombinant GST or GST-TSPYL2. GST-TSPYL2 specifically bound EZH2, as confirmed by western blot using antibodies to EZH2 to probe the enzyme complex in parallel (Fig. 1c).

**Fig. 2:**
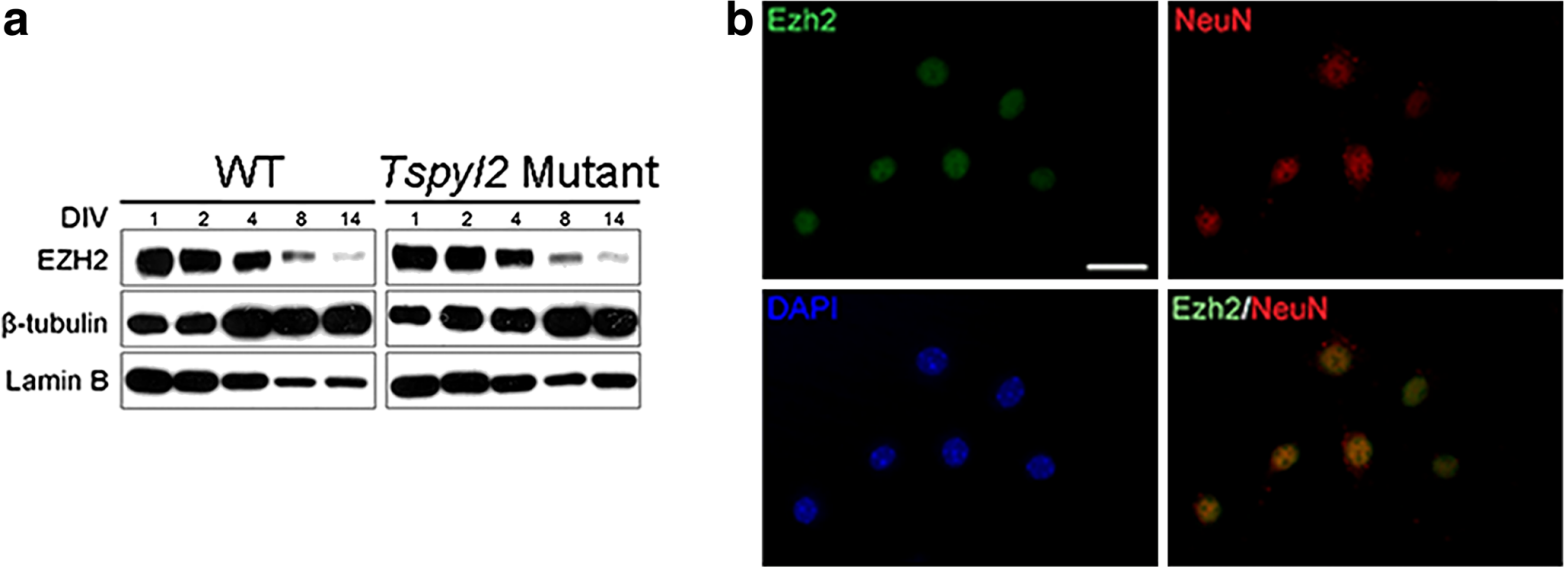
EZH2 is expressed in primary hippocampal neurons. **a** Western blot detecting EZH2 expression in primary neurons during the 14 days in vitro (DIV). Thirty μg of total protein was loaded in each lane and the nuclear cytoplasmic ratio decreased with neurite growth. **b** Immunofluorescence of EZH2 in DIV14 hippocampal neurons labeled with NeuN. WT: wild-type; scale bar = 20 μm

**Fig. 3:**
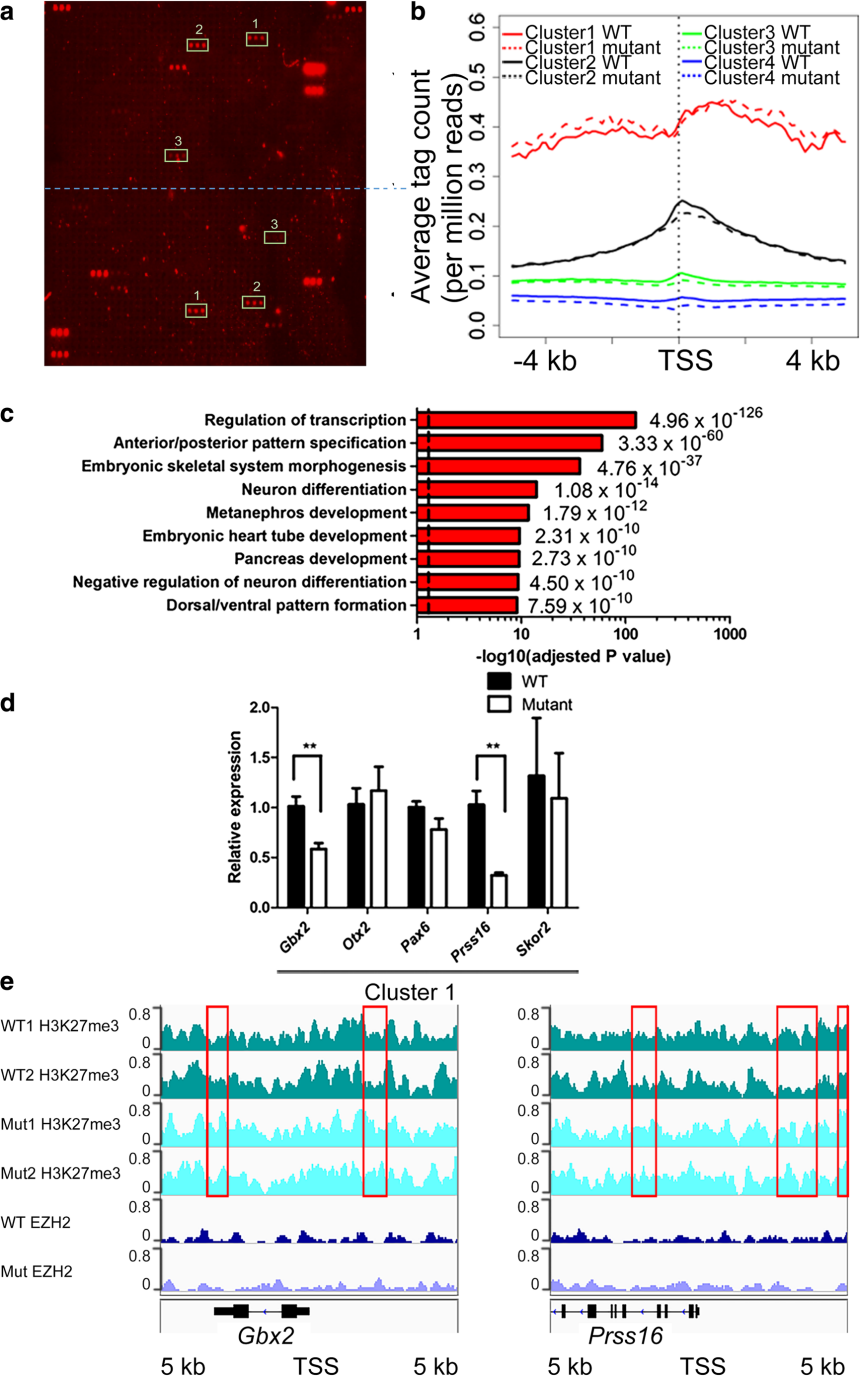
TSPYL2 regulates the level of H3K27me3 in neurons. **a** Verification of the specificity of H3K27 antibodies for ChIP-seq by hybridization to a histone peptide array. Dotted line divides the sub-arrays. Authentic positive signals appearing in triplets were boxed and peptide identities are 1, H3K27me3 + R36me2; 2, H3K27me3; 3, H4K12ac + K16 ac + H3K27me3. All unmarked triplet peptide signals are from IgG controls to indicate binding of primary antibodies and secondary antibodies. **b** ChIP-seq results of primary hippocampal neurons at 14 days in culture. H3K27 me3 occupancies at a 10-kb window centering at the TSS were classified into four clusters by k-mean clustering. Metagene analysis shows the increased level of H3K27me3 for cluster1 metagene in *Tspyl2* mutant primary neurons. **c** Selected Gene Ontology of top-enriched biological pathway annotations of cluster 1 genes (DAVID Gene Ontology Bioinformatic Resources, https://david.ncifcrf.gov/home.jsp). Dotted line, *P* < 0.05 from *P* value (a modified Fisher’s exact *P* value) adjusted by Benjamini-Hochberg correction. **d** RT-qPCR of example marked genes in cluster 1. Transcript level relative to *Hprt* and the wild-type level are set as 1. *n* = 4. Error bars represent SEM. **P < 0.01, Student *t* test. **e** Genome browser views showing ChIP-seq pileups for *Gbx2* and *Prss16*. Areas of increased H3K27me3 in mutant neurons were boxed. WT: wild-type; Mut: mutant

To identify the H3K27me3 marked genes in neurons, we employed DIV14 primary hippocampal neurons for ChIP-seq in order to minimize heterogeneity in cell types and experience-dependent variations in gene expression. The purity of neurons as determined by NeuN and DAPI staining was 95–97% throughout the 14 days culture period (not shown). We also clarified that EZH2 was expressed in primary hippocampal neurons at DIV14 by western blotting (Fig. 2a) and by co-immunostaining with the neuronal marker Neu N (Fig. 2b). For ChIP-seq, the specificity of H3K27me3 antibodies used was confirmed using a peptide array (Fig. 3a). We performed ChIP-seq in biological duplicates. To ensure sufficient sequencing depth, ~ 50 million non-duplicated reads were mapped for each sample. We did not detect any obvious changes in the genomic localization of H3K27me3 marks in neurons isolated from *Tspyl2* mutant embryos. To confirm that TSPYL2 is not generally required for the recruitment to or maintenance of EZH2 at specific target sites, we further performed ChIP-seq using ChIP-validated EZH2 antibodies with one pair of samples. We collected more than 43 million reads for each sample and identified no redistribution of EZH2 genomic binding sites (data not shown).

By k-mean clustering, we divided all RefSeq genes into four clusters from high to low modification levels based on H3K27me3 occupancy ± 5 kb of transcription start sites (TSS). Cluster 1–3 consists of 275, 1516, and 9069 genes, respectively (Online Supplemental Table). Through metagene analysis, the distribution pattern of H3K27me3 marks in the four clusters was analyzed (Fig. 3b). In general, cluster 1 metagene shows a broad peak of H3K27me3, whereas for cluster 2 and 3 metagenes, the summit is at the TSS. Cluster 4 genes are regarded as unmarked. Cluster 1 metagene is well separated from the others. The mutant showed an increase level of H3K27me3 in cluster 1 metagene, while there was a noticeable decrease in cluster 2 and 3 metagenes (Fig. 3b). Gene Ontology analysis (DAVID Gene Ontology Bioinformatics Resources, https://david.ncifcrf.gov/home.jsp) of cluster 1 genes shows that they are enriched in regulation of transcription and developmental processes, which include anterior/posterior pattern specification and neuron differentiation (Fig. 3c).

As H3K27me3 is associated with transcription repression which can be inherited during cell division [42], in mutant neurons, there should be unchanged or reduced expression of cluster 1 genes. We randomly tested several genes in cluster 1. As predicted, their expressions were low in both wild-type and mutant neurons with high threshold cycles by RT-qPCR analysis. Nevertheless, we could detect a further reduction in the expression of a few of them in mutant samples, for example *Gbx2* (*n* = 4 per genotype, *P* = 0.0095) and *Prss16* (*n* = 4 per genotype, *P* = 0.0025) (Fig. 3d). When we viewed their ChIP-seq pileups, the increase in the level of H3K27me3 in mutant neurons was only limited to small regions. The pileups for EZH2 show unremarkable variations between wild-type and mutant neurons (Fig. 3e).

### TSPYL2 Regulates the Expression of EZH2 Target Genes Important for Neuronal Functions

**Fig. 4:**
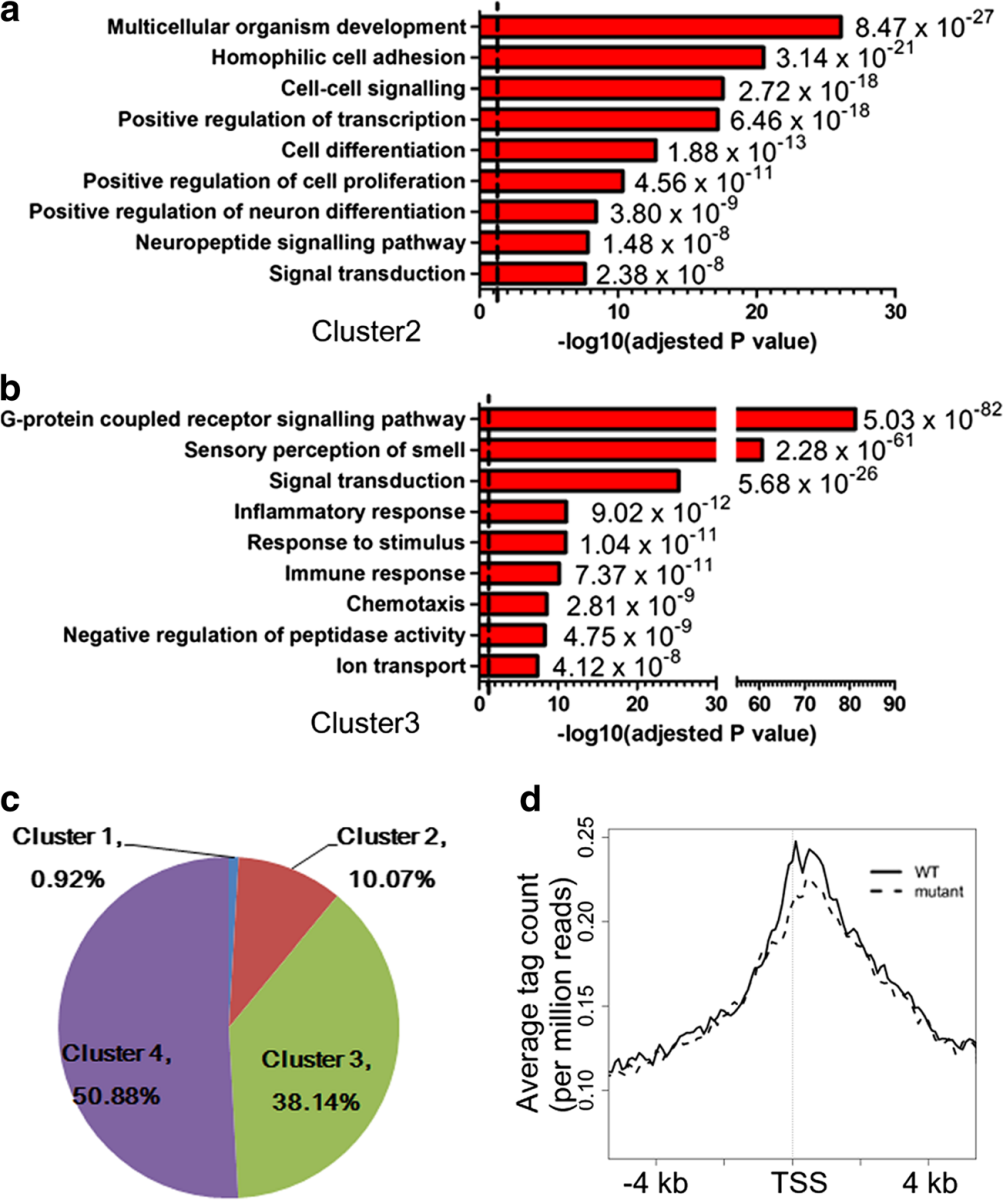
Gene Ontology analysis of genes with a low level of H3K27me3 in primary neurons. **a** Selected Gene Ontology of top-enriched biological pathway annotations of cluster 2 H3K27me3 marked genes shows enrichment in processes related to multicellular organism development (DAVID Gene Ontology Bioinformatic Resources). **b** Cluster 3 marked genes are enriched in G-protein coupled receptor signaling pathway. Dotted line, *P* < 0.05 from *P* value (a modified Fisher’s exact *P* value) adjusted by Benjamini-Hochberg correction. **c** Distribution of neuronal genes from all RefSeq genes according to the level of H3K27me3. Cluster 1 genes have the highest H3K27me3 level, while cluster 4 genes are unmarked. **d** Metagene analysis shows a peak of H3K27me3 at TSS for cluster 2 neuronal genes, which is reduced in *Tspyl2* mutant primary neurons

Gene Ontology analysis (DAVID Gene Ontology Bioinformatics Resources) of the 1516 cluster 2 genes shows that they are enriched in processes important for multicellular organism development, including positive regulation of neuron differentiation (Fig. 4a), in comparison to 9069 cluster 3 genes which are enriched in diverse pathways including G-protein coupled receptor signaling pathway, sensory perception of smell, and immune responses (Fig. 4b). Well known genes related to neuron functions, for example *Bdnf*, *Grin2a*, and *Grin2b*, are marked by H3K27me3 in mouse embryonic stem cells and primary embryonic fibroblasts [43]. To validate that neuronal genes lose this mark during neuron maturation, we further analyzed the number of neuronal genes, as defined by the annotation “synapse” or “synaptic” in Gene Ontology or “neuron” in cellular component in clusters 1–4. Of all the 2394 neuronal genes (Online Supplemental Table), 0.92, 10.07, 38.14, and 50.88% were found in clusters 1–4, respectively (Fig. 4c). We further constructed the metagene for cluster 2 neuronal genes, and it is similar to that of all cluster 2 genes. The metagene showed that *Tspyl2* knockout decreased the level of H3K27me3 around the TSS of neuronal genes (Fig. 4d).

**Fig. 5:**
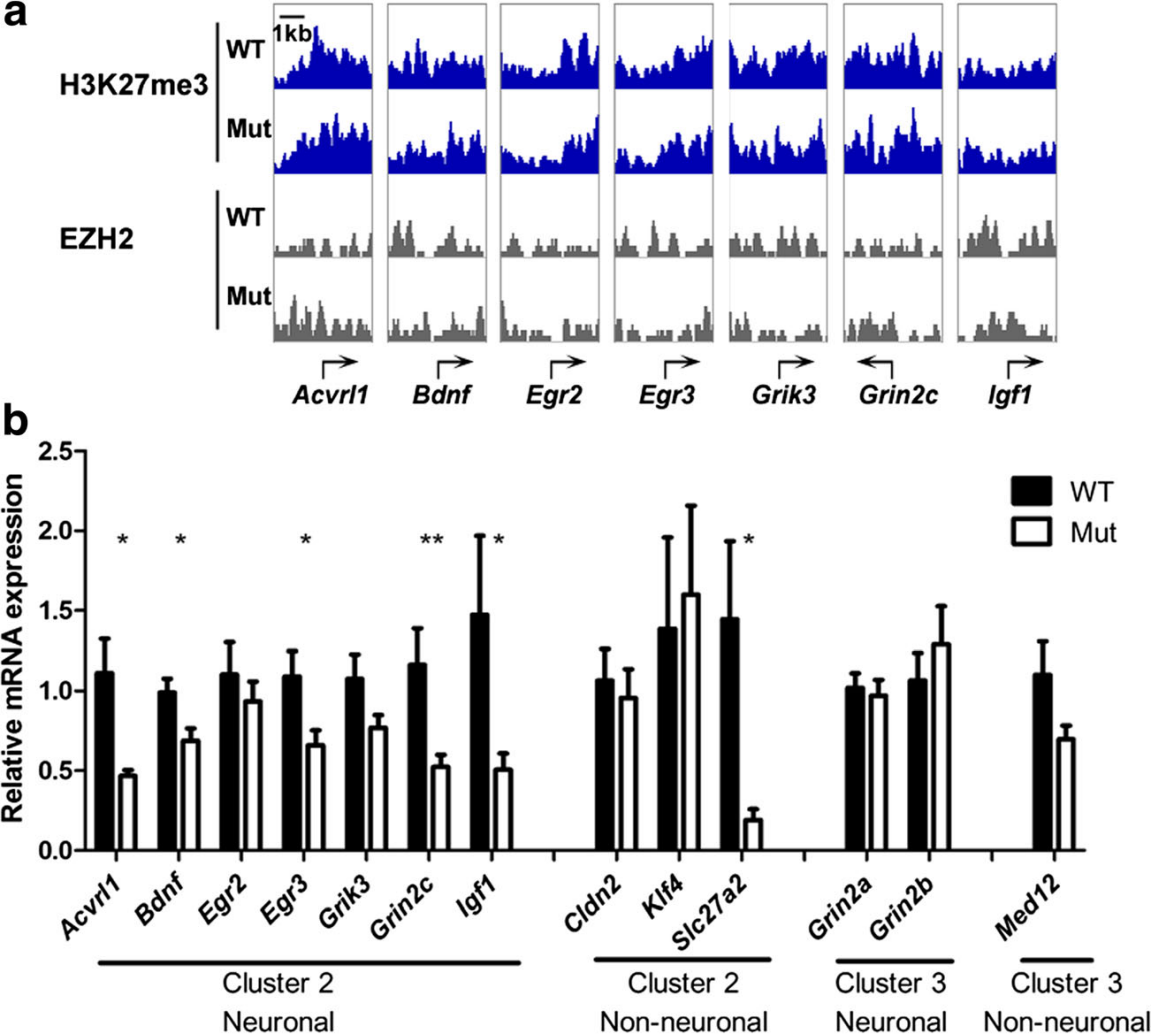
Loss of TSPYL2 reduces expression of specific H3K27me3 marked neuronal genes. **a** H3K27me3 and EZH2 ChIP-seq signals around TSS of selected genes important for neuron functions. **b** RT-qPCR showing significantly reduced expression of mainly cluster 2 neuronal genes in DIV14 mutant neurons. Transcript level relative to *Hprt* and the wild-type level was used for normalization. Error bars represent SEM. * *P* < 0.05, **P < 0.01, Student’s *t* test. WT: wild-type, *n* = 5–7; Mut: mutant, *n* = 5–11

We moved on to examine individual snapshots of well-studied neuronal genes, and these agreed with the mild differences in the metagenes (Fig. 5a). We further quantitated their expression and found reduced expression in mutant neurons in all seven neuronal genes being tested, with five genes showing statistical significant differences. These include *Acvrl1* (WT: *n* = 6; Mut: *n* = 5, *P* = 0.0243), *Bdnf* transcript variant 1, the longest transcript and therefore used for clustering (WT: *n* = 5; Mut: *n* = 5, *P* = 0.0324), *Egr3* (WT: *n* = 7; Mut: *n* = 11, *P* = 0.0234), *Grin2c* (WT: *n* = 7; Mut: *n* = 11, *P* = 0.0061), and *Igf1* (WT: *n* = 7; Mut: *n* = 11, *P* = 0.0299). By contrast, cluster 3 neuronal genes *Grin2a* and *Grin2b* did not show a difference in expression between genotypes. The non-neuronal cluster 2 gene *Slc27a2* also had reduced expression in mutant neurons (WT: *n* = 6; Mut: *n* = 6, *P* = 0.0295) (Fig. 5b).

**Fig. 6:**
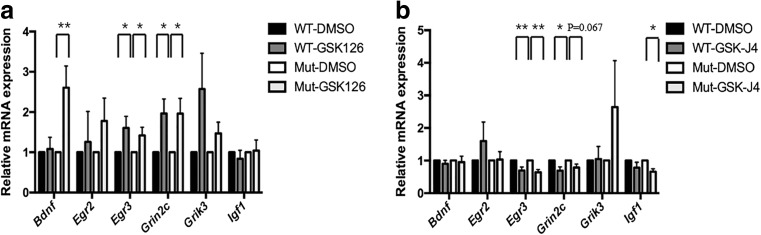
GSK126 and GSK J4 affect synaptic genes which are sensitive to *Tspyl2* knockout. **a** Effect of GSK126 on the expression of synaptic genes. *Igf1* is a neuronal but not a synaptic gene as the control. WT: wild-type, *n* = 8; Mut: mutant, *n* = 12. **b** The effect of GSK J4 on transcription of synaptic genes. WT, *n* = 9; Mut, *n* = 10. Hippocampal neurons from one embryo were seeded equally onto two wells and treated with vehicle or drug for pairwise comparison. Transcript level relative to *Hprt* and the value in the paired vehicle control was set as 1. Error bars represent SEM. * *P* < 0.05, **P < 0.01, paired *t* test

To test whether there is dynamic deposition of the H3K27me3 mark in synaptic genes, we applied GSK126, a specific inhibitor of EZH2, before we collected samples at DIV14. In both wild-type and mutant neurons, GSK126 caused a significant increase in the expression of *Egr3* (WT: *n* = 8, *P* = 0.0387; Mut: *n* = 12, *P* = 0.0429) and *Grin2c* (WT: *n* = 8, *P* = 0.0181; Mut: *n* = 12, *P* = 0.0147). Interestingly, *Bdnf* was significantly upregulated by GSK126 in mutants only (*n* = 12, *P* = 0.0053) (Fig. 6a). Conversely, pre-treatment with GSKJ4, a selective inhibitor of JMJD3 and UTX, significantly reduced the expression the *Egr3* (WT: *n* = 9, *P* = 0.0095; Mut: *n* = 10, *P* = 0.0001), and less significantly *Grin2c* (WT: *n* = 9, *P* = 0.0227; Mut: *n* = 10, *P* = 0.0670) in both genotypes (Fig. 6b).

### TSPYL2 Binds to Promoters of EZH2 Target Genes

**Fig. 7:**
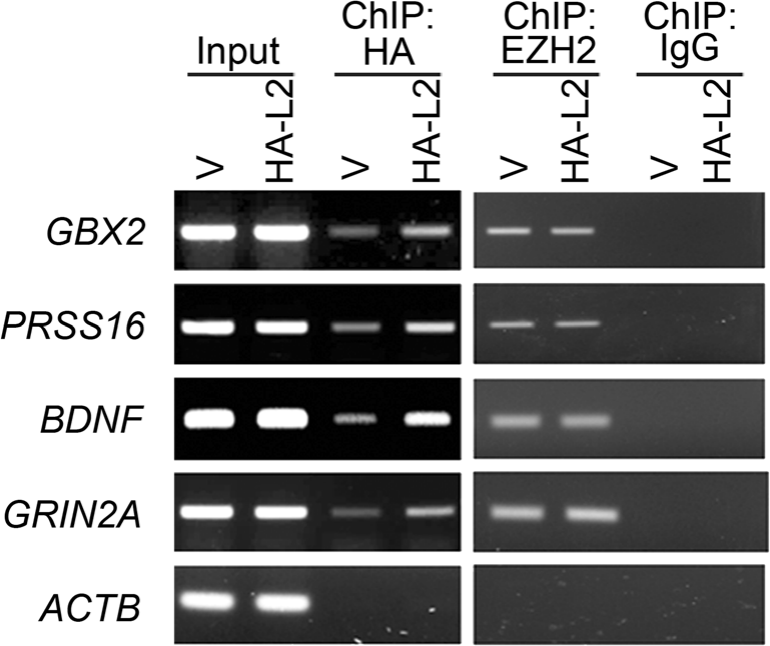
TSPYL2 and EZH2 co-exist in target promoters. Direct ChIP showing both HA-TSPYL2 and EZH2 binding at the promoter of *GBX2*, *PRSS16*, *BDNF*, and *GRIN2A*, but not the negative control *ACTB* in Be(2)-C. Cells were transfected with pcDNA3.1 (V) or expression vector for HA-TSPYL2 (HA-L2). Representative of three independent transfections

Finally, we determined whether TSPYL2 and EZH2 co-existed in target promoters. Since we could not identify TSPYL2 antibodies suitable for ChIP after an extensive search, we resolved to transfect HA-TSPYL2 into Be(2)-C human neuroblastoma cells. Cells were transfected with HA-TSPYL2 or the vector pcDNA3.1 and subject to ChIP with HA and EZH2 antibodies in parallel. As revealed in the vector control, HA antibodies gave background signals in some promoters. Nevertheless, we detected increased binding of TSPYL2 to promoters of *GBX2*, *PRSS16*, *BDNF*, and *GRIN2A* in three independent transfections. EZH2 bound equally to these regions in vector and HA-TSPYL2 transfected cells in two independent experiments (Fig. 7). The results revealed the binding of both TSPYL2 and EZH2 to the promoters of *GBX2*, *PRSS16*, *BDNF*, and *GRIN2A* in this cellular context.

## Discussion

This investigation reveals a previously unrecognized role of TSPYL2 in regulating the expression of EZH2 target genes in hippocampal neurons. Through H3K27me3 ChIP-seq, we have indirectly identified a specific subset of EZH2 target genes, which are also regulated by TSPYL2. We found that TSPYL2 interacts with EZH2 but did not affect its genomic distribution. In hippocampal neurons, some of the H3K27me3 marked genes, including *Bdnf*, *Egr3*, and *Grin2c*, are dynamically regulated by EZH2 and TSPYL2. Since *Tspyl2* loss-of-function caused reduced expression of these marked genes, TSPYL2 normally enhances the expression of these genes in differentiated neurons.

TSPYL2 interacts with transcriptional regulators such as histone acetyltransferase CBP [9], as well as histone deacetylase complexes [21] and Z3 complex [19]. In order to identify the biological relevance of these interactions, in this study, we first evaluated the effect of *Tspyl2* loss-of-function on histone modifications. Our data reveals that TSPYL2 affects the global level of H3K27 trimethylation in the hippocampus. Trimethylation of H3K27 stably repressed developmental genes in embryonic stem cells [44] and adult neurons [26]. However, a recent study also identified neuronal genes marked by H3K27me3 in neuron progenitors and post-mitotic neurons in the developing neocortex [45]. In agreement with these studies, our ChIP-seq analysis shows that genes in embryonic development and cell fate decision have the highest level of H3K27me3 mark in hippocampal neurons. These genes belong to cluster 1 in the current study and have a broad peak of H3K27me3 around the TSS. Metagene analysis shows that the level of H3K27me3 in cluster 1 genes was increased in *Tspyl2* mutant neurons. In correlation with this finding, these genes are repressed in primary neurons and further reduced expression in mutant neurons can only be detected for some genes including *Gbx2* and *Prss16*. *Gbx2* encodes a homeo-domain protein important for the development of the mid/hind brain region [46] and is transiently required for the development of specific neuron types [47, 48], whereas *Prss16* encodes a serine protease [49].

We have also clearly identified cluster 2 marked genes, which have only half or less of the level of H3K27me3 when compared to cluster 1genes, and there is a sharp peak of H3K27me3 at TSS. These genes have not only a reduced peak of H3K27me3 but also reduced transcript levels in mutants. We propose that TSPYL2 is recruited to these promoters through interaction with the PRC2 complex. The interaction may be strengthened by REST, which has been shown to recruit PRC2 complex to a subset of neuronal genes in mouse embryonic stem cells [33]. Furthermore, TSPYL2 is an interacting partner of REST [21]. TSPYL2 could remain in these promoters and is involved in subsequent gene activation.

We moved on to test the importance of EZH2 and TSPYL2 in the regulation of well-known synaptic genes. Treatment with selective inhibitors of EZH2 and H3K27 demethylases confirmed that synaptic genes perturbed in *Tspyl2* mutant neurons (*Bdnf*, *Egr3* and *Grin2c*) were upregulated by inhibition of EZH2, while synaptic genes unaffected in mutant neurons (*Egr2* and *Grik3*) were not sensitive to such treatment. Collectively, the data strongly suggest that TSPYL2 and EZH2 dynamically regulated the expression of important synaptic genes in hippocampal neurons. Defects in the fine tuning of gene expression can contribute to the phenotype of our *Tspyl2* knockout mice reported previously [9, 10]. For example, *Egr3* is important for hippocampal long-term potentiation and fear conditioning [50]. Both of these processes are impaired in our *Tspyl2* mutant mice [9]. The regulation of *Bdnf* is complex and involves multiple splice variants [37, 51]. REST, EZH2, JMJD3, and CBP have all been reported to regulate the transcription of *Bdnf* [36, 37, 52]. Using our simplified culture model, there is impaired transcription of *Bdnf* exon 1 in the *Tspyl2* mutant neurons which can be rescued by inhibiting EZH2. Specifically, increase in the transcription of *Bdnf* exon 1 has been reported after fear conditioning and its extinction [53–55].

**Fig. 8:**
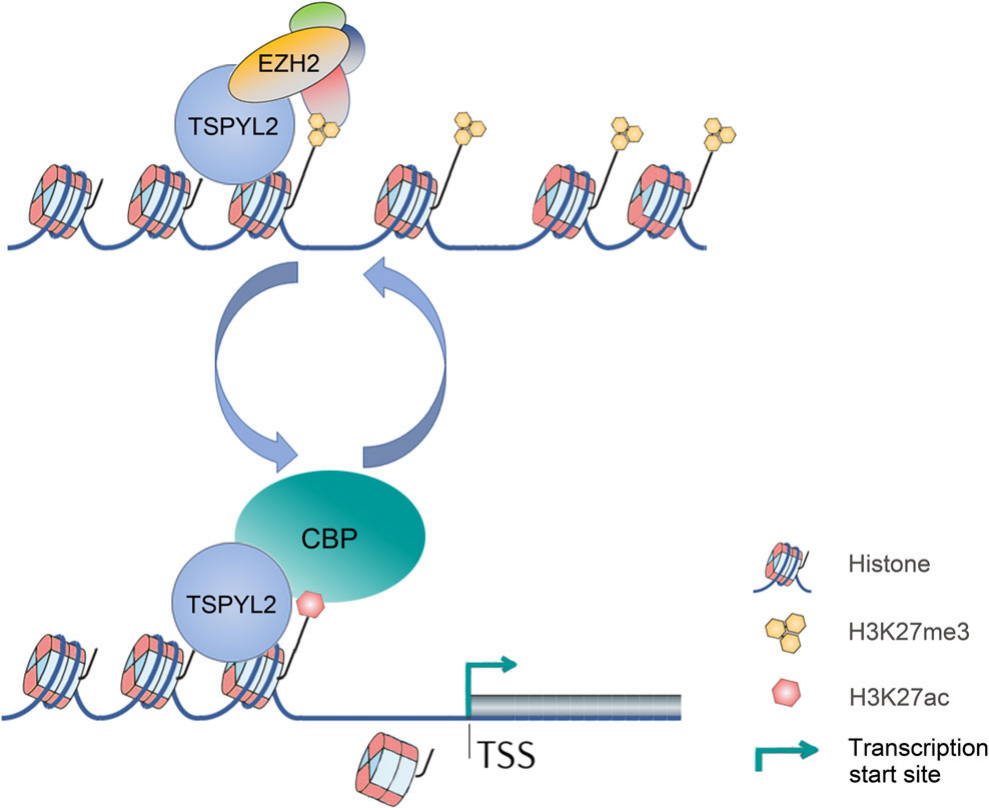
Model of TSPYL2 in transcription regulation of neuronal genes

The mild changes in H3K27me3 level, but significantly reduced gene expression in mutant neurons, suggest that TSPYL2 is involved in multiple steps in regulating gene expression. As NAPs can tether the transcription regulatory complex to chromatin, a possible explanation is the impaired dynamic localization of EZH2 and other transcriptional regulators, such as p300 and CBP, in the absence of TSPYL2 at target promoters. Highlighting the dynamic and transient nature of protein interactions in transcription regulation, RbAp46/48, a core component of the PRC2 complex, also interacts with CBP and enhances its activity. Importantly, impairment in this process can lead to memory-loss during aging [56]. Interestingly, we also identified RbAp48 as a weak TSPYL2 interacting protein in a pull-down assay using HEK293 cells (not shown), confirming the findings in carcinoma cells [21]. To account for the reduced expression of synaptic genes such as *Egr3* and *Grin2c* in *Tspyl2* mutants, our current model is that TSPYL2 is recruited to promoters of neuronal genes via the EZH2 enzyme complex. Upon gene activation, TSPYL2 remains as a bridge between histone and the new transcription regulatory complex. Upon histone acetylation, TSPYL2 may also play a role in nucleosome eviction, which facilitates transcription initiation (Fig. 8).

In summary, our data show that TSPYL2 and EZH2 are involved in the dynamic regulation of the expression of a number of neuronal genes. Specifically, loss of *Tspyl2* impaired the expression of these important genes including *Bdnf*, *Egr3*, and *Grin2c* in hippocampal neurons. Even though the exact target genes might vary as neurons continue to mature post-natally, our work provides a proof of principle study to show that TSPYL2 epigenetically regulates gene expression in neurons through multiple interacting partners. This fine tuning of gene expression provides an attractive mechanism of how *TSPYL2* contributes to intellectual performance and neurodevelopmental syndromes in humans.

Accession number: All raw sequencing data and normalized tag pileup files can be accessed from GEO Series entry GSE74733. (https://www.ncbi.nlm.nih.gov/geo/query/acc.cgi?acc=GSE74733)

## Electronic Supplementary Material

ESM 1 (XLSX 540 kb)
